# Interpreting diagnosis outcomes for tuberculosis to timely and reliably predict non-tuberculosis mycobacteria isolation

**DOI:** 10.1099/jmm.0.002009

**Published:** 2025-04-28

**Authors:** Biyi Su, Haoran Qi, Yaoju Tan, Hairong Huang

**Affiliations:** 1 Clinical Laboratory, Guangzhou Chest Hospital, Guangzhou, PR China; 2 National Clinical Laboratory on Tuberculosis, Beijing Key Laboratory for Drug-Resistant Tuberculosis Research, Beijing Chest Hospital, Capital Medical University, Beijing Tuberculosis and Thoracic Tumor Institute, Beijing, PR China

**Keywords:** accuracy, differential diagnosis, indicator, non-tuberculous mycobacteria (NTM), tuberculosis (TB)

## Abstract

**Introduction.** Timely distinguishing non-tuberculous mycobacteria (NTM) from *Mycobacterium tuberculosis* is needed, but it is challenging.

**Hypothesis.** Smear-positive and tuberculosis (TB) molecular-test-negative outcomes could timely and accurately predict NTM existence in the clinical specimen.

**Methodology.** Laboratory outcomes of the smear test and TB molecular test outcomes were evaluated in a high TB and NTM prevalence setting. Additionally, the interferon-gamma release assay (IGRA) outcome was scrutinized to assess its supplementary value to the above strategy.

**Results.** The smear-positive/Xpert MTB/RIF (Cepheid, USA) outcomes accurately predicted 91.67% (198/216) of the NTM isolation, while that of smear-positive/Simultaneous Amplification and Testing method (SAT-TB) (Rendu Biotechnology, China) negative outcomes was 84.5% (169/200). Applying these indicators to rule out TB could achieve an accuracy of up to 99.49% (3435/3453). Combining smear-positive, Xpert-negative and SAT-TB-negative outcomes increased the accuracy up to 95%. Adding a negative IGRA outcome to the indicators further increased the accuracy to over 96%, albeit at the cost of losing prediction sensitivity. When evaluating the strategy in NTM isolates, the indicators successfully predicted about 40% of these isolations with over 92% accuracy.

**Conclusion.** A smear-positive/molecular TB test-negative outcome could timely and accurately predict NTM isolation in the given setting. This strategy could predict ~40% of the NTM isolations of the patients on their first day of hospital visit.

## Data Availability

All the data included in the manuscript are available to the public.

## Introduction

With the escalating global prevalence, non-tuberculous mycobacterial (NTM) disease poses a challenge to global tuberculosis (TB) control efforts [1]. NTM comprises a diverse group of mycobacteria distinct from the *Mycobacterium tuberculosis* (Mtb) complex and *Mycobacterium leprae*, and is widely present in the surroundings. Individuals with compromised immunity have a higher chance of contracting NTM disease [2,3], but an increasing number of cases without known immune deficiencies have been reported [4]. In recent years, NTM isolations and diagnosed cases have substantially increased worldwide [5,6]. Due to the similarity in bacterial features between NTM and Mtb, and the resemblance in symptoms and radiological images of the diseases they cause, NTM lung disease is easily misdiagnosed as TB, leading to mistreatment and delayed treatment [7]. With an intrinsic resistance to many anti-TB drugs, numerous NTM cases have been mismanaged as drug-resistant TB for a considerable duration before receiving an explicit diagnosis [8]. NTM disease has been particularly overlooked in high-TB burden countries. The main reason lies in the fact that no feasible NTM identification method is available. Given the significantly lower prevalence of NTM compared with TB [9,10], routine differential diagnosis between them is deemed less cost-effective.

Due to the country’s vast expanse and distinct geographical and climatic features in various regions, NTM disease prevalence rates vary significantly nationwide [11]. In our routine algorithm for diagnosing pulmonary TB, we observed that 95.5% of enrolled patients with synchronously positive smear test results but negative Xpert MTB/RIF assay (Cepheid, USA) outcomes were later identified as having NTM isolation [12,13]. Furthermore, when a negative interferon-gamma release assay (IGRA) outcome was added in the combination above, the prediction accuracy was achieved 100%, but at a great loss of prediction sensitivity. Since this previous study was conducted in a high TB but low NTM prevalence setting, we are eager to know how this strategy works in a high TB and high NTM prevalence setting. Guangdong province reports the highest NTM prevalence in China [14], where there are significant demands in distinguishing between TB and NTM diseases in routine work. Additionally, we also evaluated another Chinese domestic molecular testing method with the same strategy. Simultaneous amplification and testing method for the detection of Mtb (SAT-TB) (Shanghai Rendu Biotechnology Co., Ltd., Shanghai, China) targeting the 16s RNA fragment of Mtb is used for TB diagnosis [15]. In clinical practice, it is about 10%–15% less sensitive when compared with the Xpert assay. We like to know how the prediction strategy works with molecular tests other than Xpert assay with different sensitivities to find out if this strategy could be easily extrapolated to other molecular TB tests.

## Method

### Study design

A retrospective study was conducted at Guangzhou Chest Hospital in Guangdong Province, the largest designated TB hospital located in southeast China. Based on routine laboratory data, NTM comprised ~30 % of all mycobacterial isolates. All data were retrieved from the laboratory information system.

### Investigation of the smear-positive and molecular-test-negative data for concept validation

Between January 2020 and September 2022, patients with a new episode of pulmonary disease were investigated. Those who underwent the focal tests for the first time in the hospital or at least 1 year from their last examinations were enrolled. Patients with smear-positive outcomes were then followed up for simultaneous Xpert assay and/or SAT-TB assay outcomes. For patients who had more than one smear test performed within a week, any positive smear test was considered. If a patient had more than one Xpert assay or SAT-TB assay performed within the period, then all the Xpert outcomes or SAT-TB assay results must be negative. All patients with smear-positive but molecular-test-negative outcomes were subsequently followed up for simultaneous culture and species identification outcomes, as well as IGRA results [16].

### Investigation of the clinical NTM strains for concept validation

All the NTM strains isolated in 2019 were included in the investigation, and their additional laboratory examination outcomes, such as smear test, Xpert assay, SAT-TB assay and IGRA, which were performed contemporaneously with the focal culture, were collected.

## Laboratory testing

### Smear testing

A direct smear was prepared and stained with auramine followed by examination using light-emitting diode microscopy (Carl Zeiss AG, PrimoStar, Germany).

### MGIT960 culture and MPT64 antigen assay

The MGIT960 culture was conducted following the manufacturer’s instructions (Becton, Dickinson and Co, USA). In vials that showed a positive signal, acid-fast bacilli (AFB) were observed through microscopic examination. The broth from the positive vial was tested using an MPT64 antigen detection kit (Genesis Corporation, Hangzhou, China) [17].

### Species/subspecies identification by DNA microarray chip method

The isolate was identified at the species level by using the Mycobacterial Species Identification Array Kit (CapitalBio Technology Inc., Beijing, China) [18].

### Xpert MTB/RIF assay, SAT-TB assay and IGRA

Xpert MTB/RIF assay, SAT-TB assay and IGRA were conducted following the manufacturer’s instructions [13,15, 16].

## Data management

Sensitivity was defined as the proportion of patients who yielded a smear-positive/molecular-test-negative outcome among all NTM-isolated patients. The specificity was defined as the proportion of patients with TB who underwent both tests but did not produce a smear-positive/molecular-test-negative outcome. The accuracy of the indicator was defined as the proportion of patients who proved to have NTM isolation among those who yielded a smear-positive/molecular-test-negative outcome. The chi-squared test was performed using SPSS version 19.0 (IBM, Armonk, NY, USA) to compare the prediction accuracy of different combining strategies, with differences considered statistically significant at *P*<0.05.

## Results

### The patient had the focal test performed

**Fig. 1.**
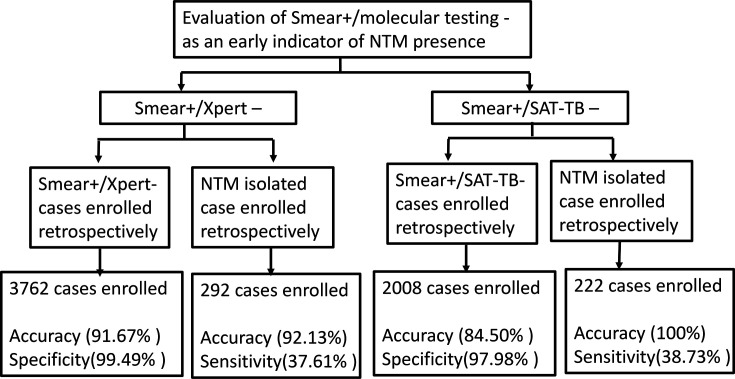
Summary of the study design and the main findings. The accuracy of the indicators of NTM existence was analysed in two routes: (1) Began from the patient who performed smear test; (2) Began from the patient who had NTM isolated. The performances of different routes regarding different molecular tests were summarized in the bottom boxes.

During the study period, a total of 66,574 possible patients with TB underwent 2,21,911 smear tests, with 9,625 (14.45 %) yielding positive smear outcomes. Among these smear-positive patients, 3,762 underwent Xpert assay, and 2,008 underwent SAT-TB assay during the corresponding episode (Fig. 1).

### The follow-up data of the patient with smear-positive but Xpert-negative outcome

Out of the 3,762 smear-positive cases that also underwent the Xpert assay, 8.70% (327/3,762) showed negative results. Subsequently, these patients were examined for their respective culture outcomes. Due to either the absence of culture, culture negativity or the lack of species identification with the isolates, only 216 patients were eligible for analysis. Among them, 198 patients were ultimately found to have NTM isolation, and 18 (8.33 %) were Mtb according to the MPT64 antigen test and species identification. Therefore, the accuracy of using smear-positive/Xpert-negative outcome to predict NTM isolation was 91.67% (198/216). Among the smear-positive patients with TB, the specificity of this indicator reached ~99.49% [3435/(3435+18)] (Figs1  2).

**Fig. 2.**
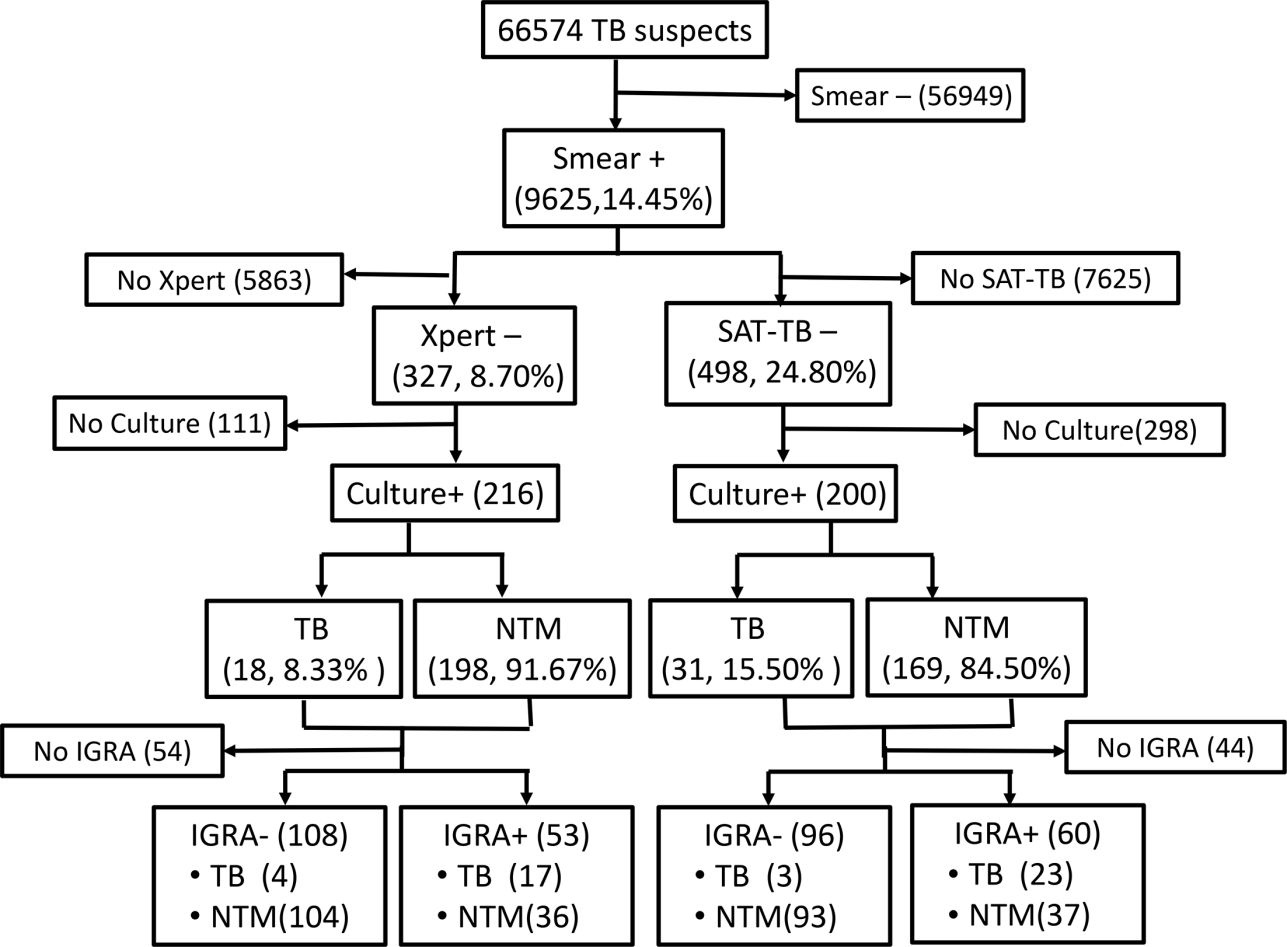
Flow chart of patient enrolment and outcomes. Detailed laboratory examination data of the enrolled patients who performed the required tests and the outcomes.

### The follow-up data of the patient with smear-positive but SAT-negative outcome

Out of the 2,008 smear-positive cases that also underwent the SAT-TB assay, 24.80% (498/2,008) had negative results. A total of 200 patients were culture-positive and had species identification outcomes as well. Among them, 169 patients were finally categorized as having NTM isolates, while the leftover 31 patients were identified as TB. Therefore, the smear-positive and SAT-TB negative outcome had 84.5 % (169/200) accuracy in predicting the NTM presence in the sputum specimen (Figs1  2). SAT-TB assay as the focal molecular test demonstrated lower accuracy than the Xpert assay (χ^2^=5.133, *P*<0.023). Among the smear-positive patients with TB, the specificity of this indicator was ~97.99 % [1510/(1510+31)], which is also lower than the Xpert assay (χ^2^=24.361, *P*=0)

### The prediction accuracy when combining smear-positive, Xpert-negative and SAT-TB-negative

Out of the 180 smear-positive/Xpert-negative cases that also had SAT-TB-negative outcomes, 171 were identified with NTM isolates. Therefore, the accuracy of using smear-positive/Xpert-negative/SAT-TB-negative outcome as an indicator of NTM isolation was 95% (171/180). Among the 175 smear-positive/SAT-TB-negative cases that also had Xpert-negative outcomes, 163 exhibited NTM isolation. Hence, the accuracy of using smear-positive/SAT-TB-negative/Xpert-negative as an indicator of NTM isolation was 93.14% (163/175).

### The prediction accuracy of smear-positive/molecular-test-negative outcome when adding additional IGRA-negative outcome

Among the 216 smear-positive/Xpert-negative patients, 161 underwent IGRA testing in this episode and obtained eligible outcomes, of which 53 were positive and 108 were negative. Among the 108 smear-positive/Xpert-negative/IGRA-negative cases, 104 were confirmed to have NTM isolates. Therefore, the accuracy of using smear-positive/Xpert-negative/IGRA-negative as an indicator of NTM isolation was 96.30% (104/108). However, in the 53 smear-positive/Xpert-negative/IGRA-positive patients, 17 were identified as having TB, and 36 as having NTM isolations. Integrating IGRA results into the indicator group increased the accuracy of NTM isolation prediction by 9.34% [from 86.96% (140/161) to 96.30 % (104/108)], but lost sensitivity by 25.71% [36/(104+36)].

Among the 200 smear-positive/SAT-TB-negative patients, 156 underwent IGRA testing in this episode and obtained eligible outcomes, of which 60 were positive and 96 were negative. Among the 96 smear-positive/SAT-TB-negative/IGRA-negative cases, 93 were conclusively identified as having NTM isolations. Hence, the accuracy of using smear-positive/SAT-TB-negative/IGRA-negative outcome as an indicator of NTM isolation was 96.88% (93/96). However, in the 60 smear-positive/SAT-TB-negative/IGRA-positive patients, 23 were identified as having TB, and 37 as having NTM isolations. Integrating IGRA results into the indicator group increased the accuracy of NTM isolation prediction by 12.35% [from 84.5%(169/200) to 96.88% (93/96)], but lost sensitivity by 28.46% [37/(93+37)].

### The smear and molecular testing outcomes of patients who had NTM isolated during the disease differentiating diagnosis stage

In 2019, a total of 292 cases that met the requirements had NTM strains isolated. Among them, eight who had a mixture of Mtb and an NTM strain in the same specimen were excluded from further analysis. Of the remaining 284 cases, 218 cases underwent a smear test and Xpert assay at the time of the disease diagnosis stage. Of these, 89 (40.83 %, 89/218) were smear-positive. Therefore, in these smear-positive and NTM-isolated cases, smear-positive/Xpert-negative results predicted 92.13% (82/89) of NTM isolation. Overall, the sensitivity of smear-positive/Xpert-negative as an indicator of NTM presence was 37.61%(82/218) (Figs1  2).

A total of 222 of these NTM-isolated cases underwent a smear test and SAT-TB assay at the time of the disease diagnosis stage, of which 86 (38.74%, 86/222) were smear-positive. Surprisingly, all (100%, 86/86) of these smear-positive cases had negative concurrent SAT-TB test outcomes. SAT-TB assay showed higher prediction sensitivity than the Xpert assay in the combining strategies (χ^2^=6.386, *P*=0.012). Overall, the sensitivity of smear-positive/SAT-TB-negative as an indicator of NTM presence was 38.73% (86/222).

### NTM species distribution

**Fig. 3.**
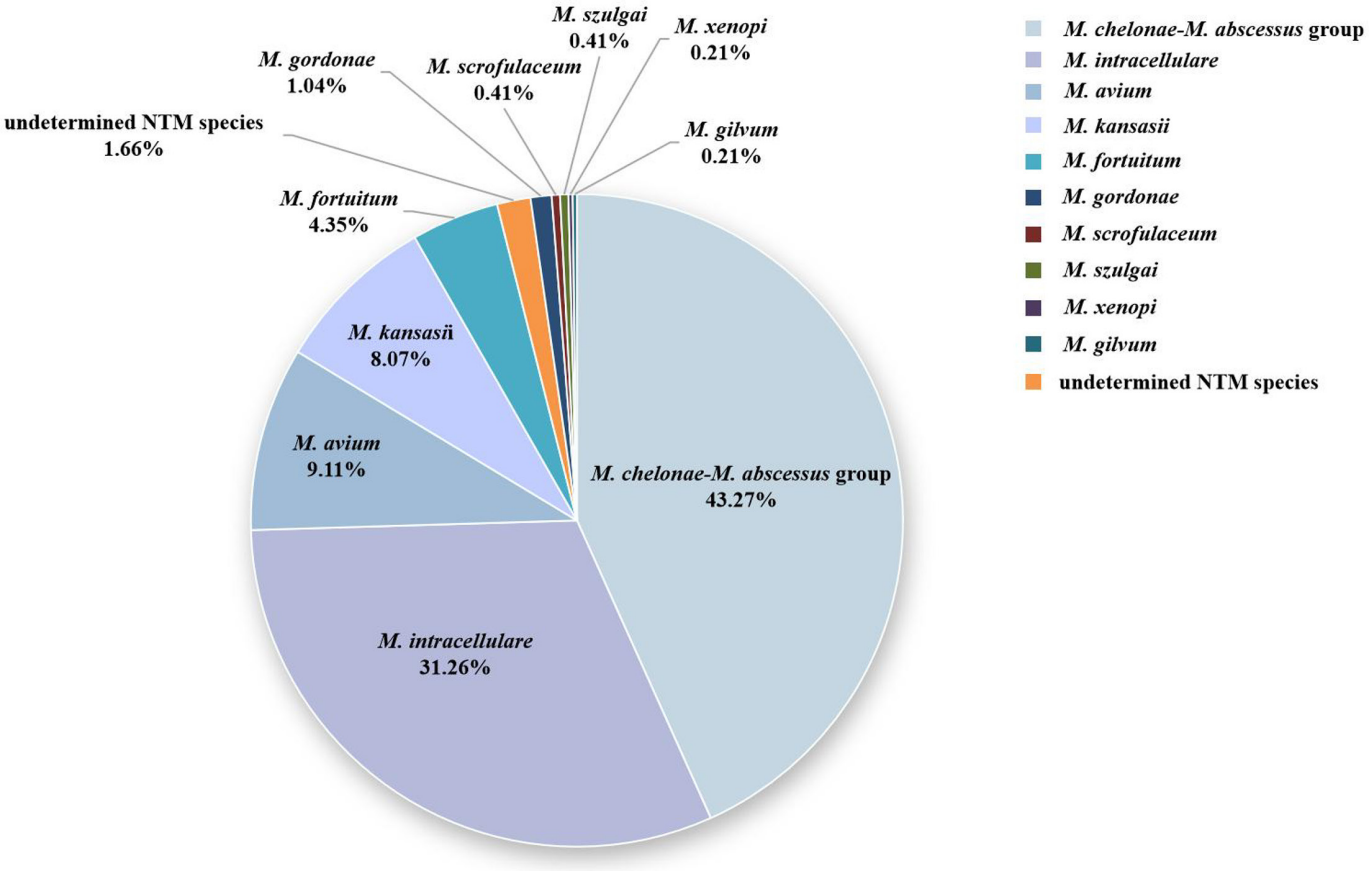
The species constitution of 483 clinical NTM isolates.

In this study, a total of 483 NTM strains were recruited, comprising both the follow-up study of the indicators and the analysed isolated NTM strains. The distribution of different species is illustrated in Fig. 3. The *M. chelonae–M. abscessus* group constituted 43.27% of all these NTM strains (the method could not separate these two species), followed by *M. intracellulare* (31.26%), *M. avium* (9.11%) and *M. kansasii* (8.07%). The prediction accuracy of the indicators showed no significant difference when analysed in different NTM species separately (data not shown).

## Discussion

Smear testing is universally applied for TB diagnosis due to its simplicity, affordability and rapidity. However, its notorious drawback is the low sensitivity, with a limit of detection (LOD) exceeding 5,000 AFB ml^−1^ for fluorescence microscopy and >10,000 AFB ml^−1^ for light microscopy [19]. Furthermore, smear tests cannot differentiate between Mtb, NTM and other non-mycobacterial acid-fast bacteria. In the last decade, molecular tests, such as Xpert MTB/RIF, have become the initial choice for TB diagnosis due to their high sensitivity and point-of-care test characteristics. The LOD of the Xpert assay is reported as 131 AFB ml^−1^, with a turnaround time of about 2 h [20]. A systematic review reported over 97% sensitivity for Xpert assay in smear-positive TB cases, with specificity exceeding 99% [21]. Based on these aforementioned facts, we propose that a smear-positive/Xpert-negative outcome for a patient could very possibly indicate the presence of NTM. Additionally, according to the rationale of IGRA, a positive outcome generally indicates TB infection, whereas the majority of NTM species do not have overlapping immunity reactions with the TB-specific antigens included in IGRA. Therefore, smear positive and IGRA negative might also suggest an NTM infection. These hypotheses were well proved in our previous study conducted in a hospital located in southern China, [13] and in this study, they are further examined in a different setting and with other TB molecular tests.

Compared with TB, molecular tests targeting NTM identification are scarce in China. In this focal hospital, Mtb and NTM differentiation is initiated by an MPT64 antigen test when an isolate is cultivated, which usually takes place 1–3 weeks after sample processing. In this study, by referencing the routine laboratory outcomes of patients, typically obtainable on the first few days of the hospital visits, 91.67% (198/216) of cases yielding smear-positive/Xpert-negative results were later found to have NTM isolations and 84.5 % (169/200) for SAT-TB. The application of the Xpert assay as the focal molecular test demonstrated significantly better performance than the SAT-TB assay (χ^2^=5.133, *P*<0.023). This discrepancy might be attributed to the higher sensitivity of the Xpert assay compared with the SAT-TB assay [22]. Integration of these two molecular tests, the smear-positive/Xpert-negative/SAT-TB-negative outcome, resulted in increased prediction accuracy (over 93%). Therefore, we presume that other molecular tests with good performance could be applicable as well. Furthermore, when integrated with IGRA-negative results into the smear-positive and molecular-test-negative combination, both indicators achieved significantly better accuracy (96.30% and 96.88%, respectively). However, this improvement comes at the cost of decreased sensitivity in prediction (25.71% and 28.46%, respectively). The main reason lies in the fact that China is a high TB burden country with a high latent TB rate of ~20% [23]. We reasonably presume that in low TB burden countries, smear-positive and IGRA-negative results might also be a sensitive and reliable indicator of NTM isolation.

In this study, ~40% of the NTM isolated cases were smear-positive, and 92.86% (91/98) of them had negative Xpert results, while 100% (86/86) had negative SAT-TB outcomes. Although SAT-TB outperformed the Xpert assay in this analysis, five out of the seven NTM isolated cases with Xpert-positive outcomes were ultimately diagnosed as having TB as well. The good performance of the SAT-TB assay here might also reflect a less sensitive feature of the test. A mixed infection of Mtb and NTM strains may impact the prediction accuracy. Additionally, even though we focused on tests performed within a few days only, the bacilli might be unevenly distributed in these specimens, potentially causing some misjudgments. Overall, in this hospital, applying a smear-positive/molecular-test-negative outcome as an early indicator of NTM isolation could achieve about 40% sensitivity in predicting NTM isolation, with accuracy at about 90%.

The limitations of this study should be noted. First, this study was conducted in a single centre. The rationale of the study and other molecular diagnostics should be evaluated in different health centres to better interpret this rapid, simple and practical prediction strategy. Secondly, NTM isolation is not necessarily suggestive of disease and may sometimes indicate respiratory tract colonization or contamination [24,25]. Our study did not differentiate these different conditions, but a smear-positive outcome with NTM had a good chance of being a real NTM disease case [26].

## Conclusions

In summary, a smear-positive outcome combined with a negative TB molecular test is a reliable indicator of the presence of NTM in the given settings. The addition of a negative IGRA outcome further enhanced the accuracy of the prediction. With this strategy, the identification of NTM could be expedited from weeks to the first day of hospital visits for smear-positive NTM patients.

## Abbreviations

AFB: acid-fast bacilli
IGRA: interferon-gamma release assay
LOD: limit of detection
NTM: non-tuberculous mycobacteria
SAT-TB: simultaneous amplification and testing method for detection of the Mtb
TB: tuberculosis
